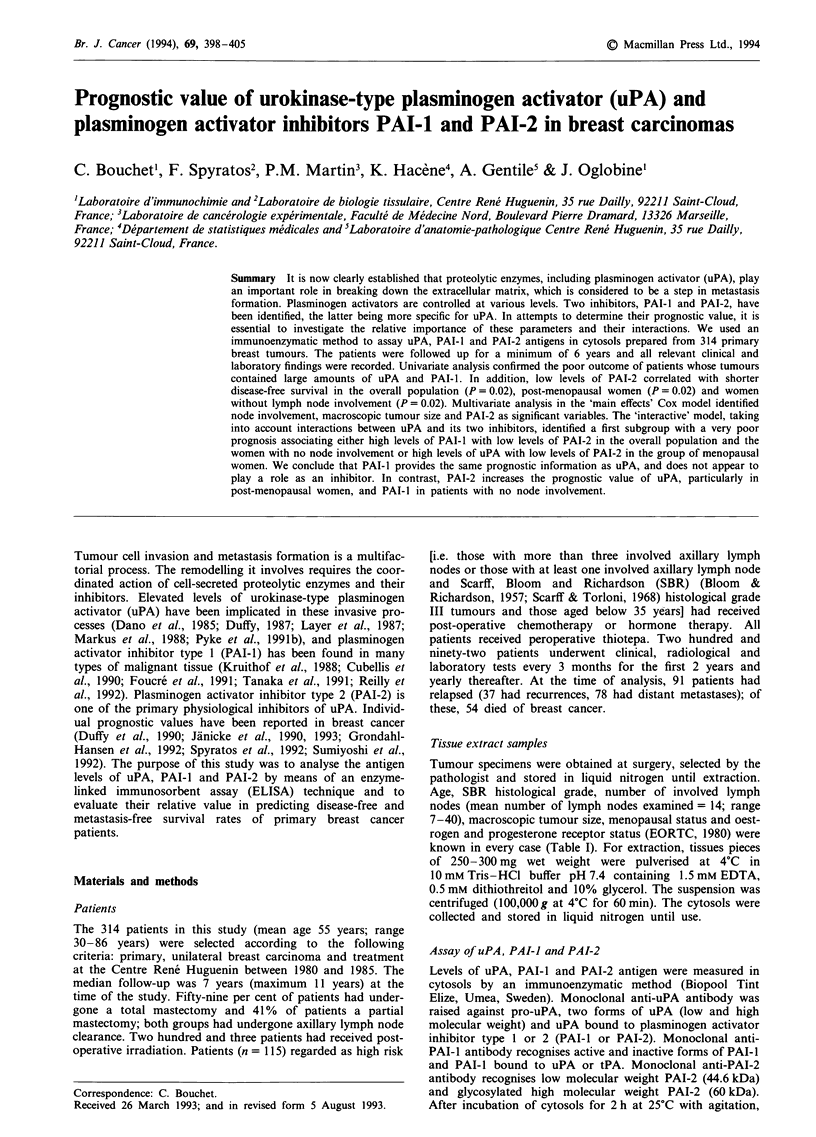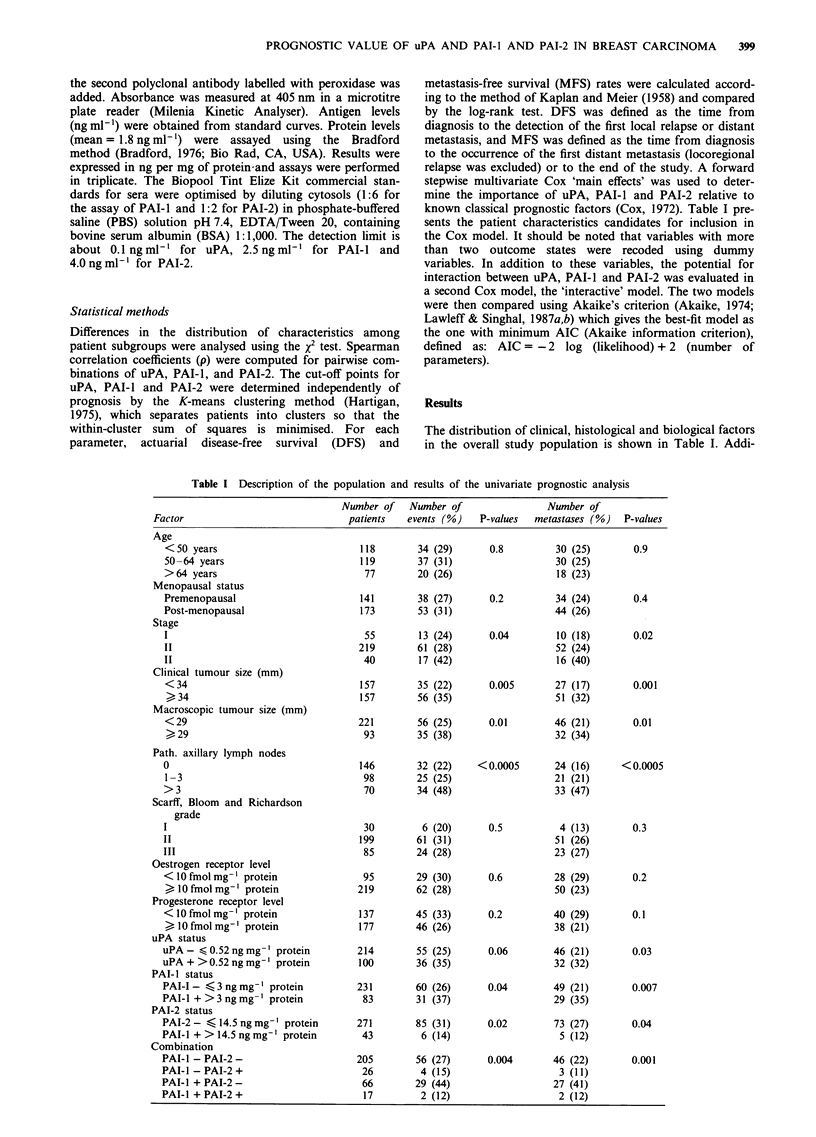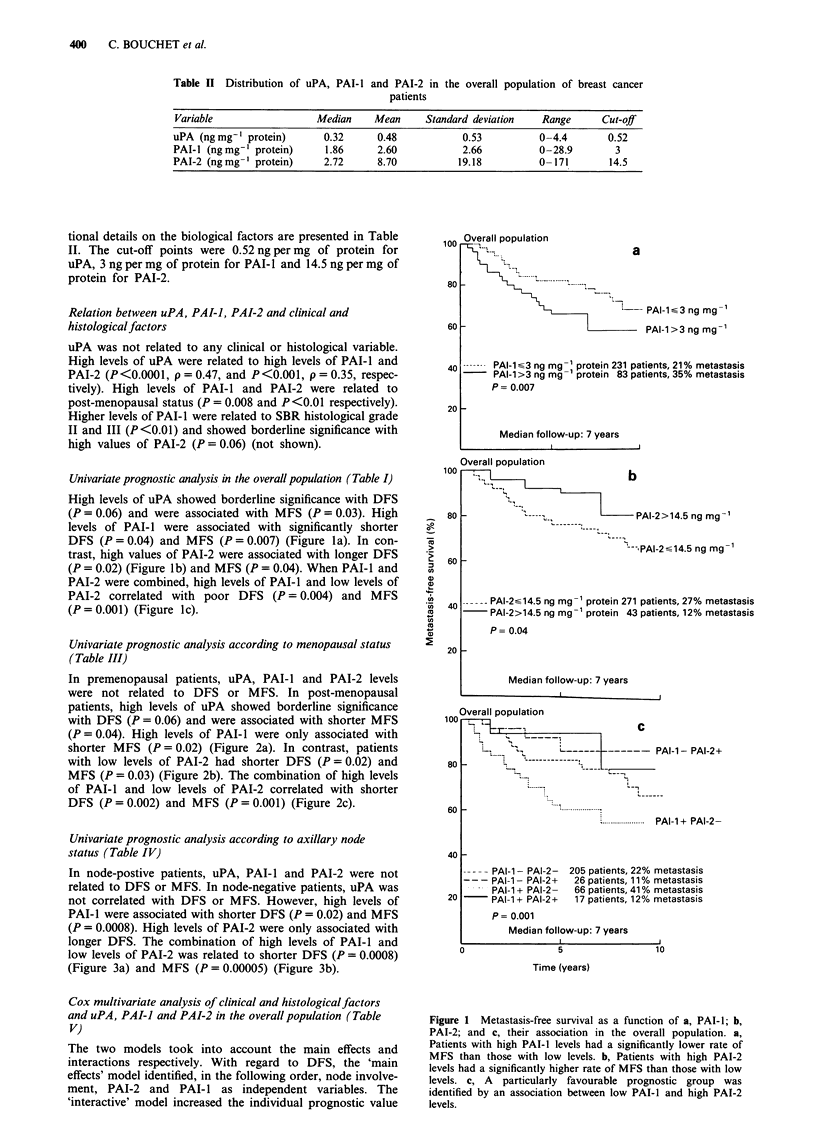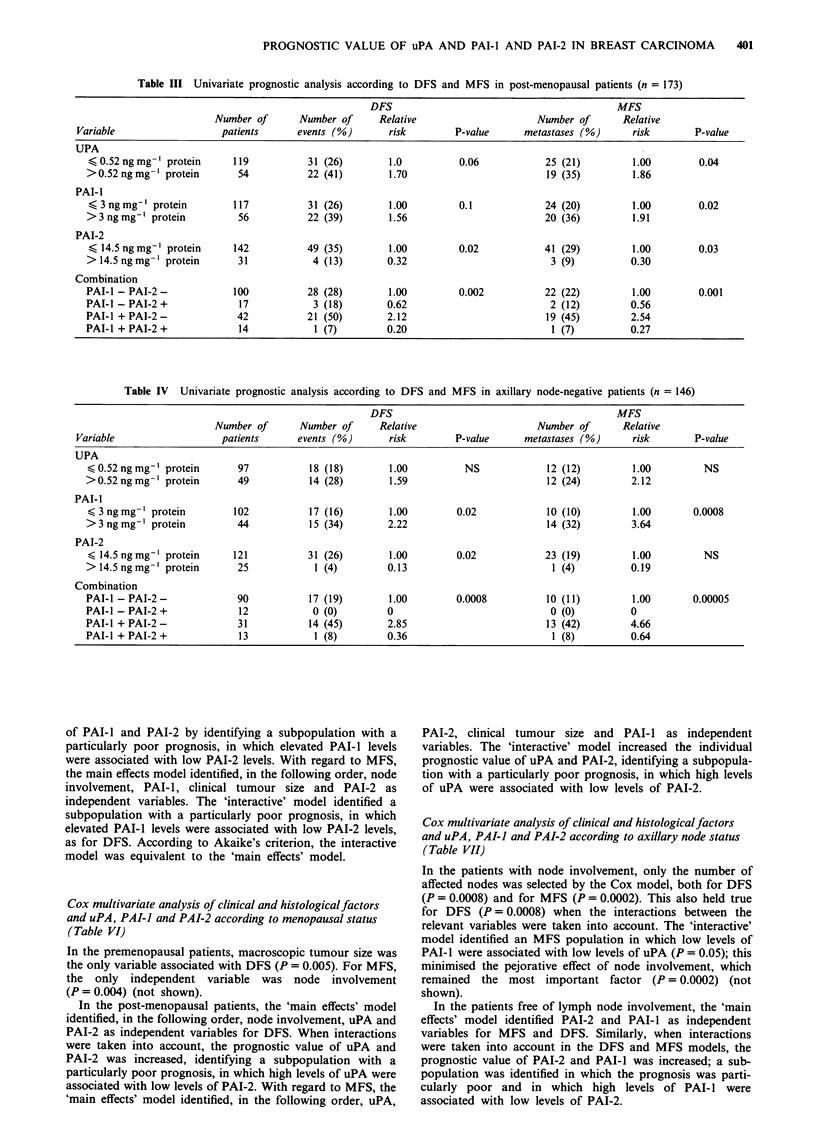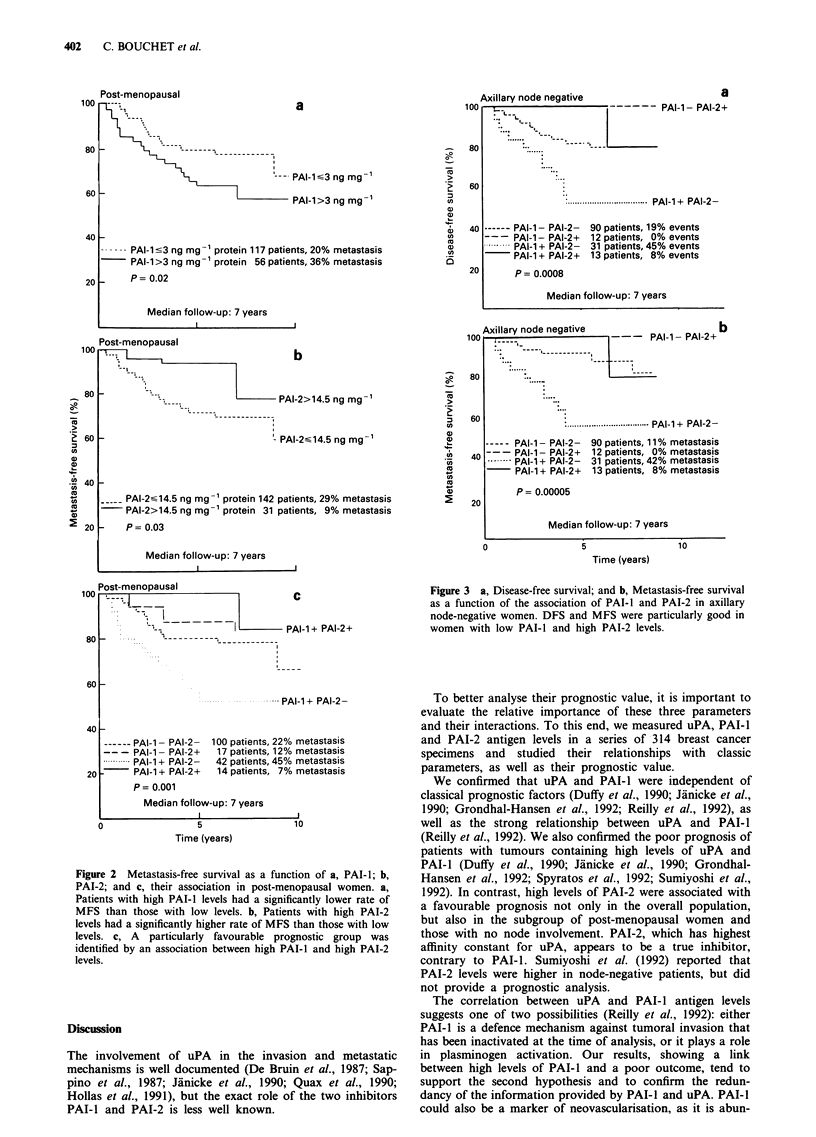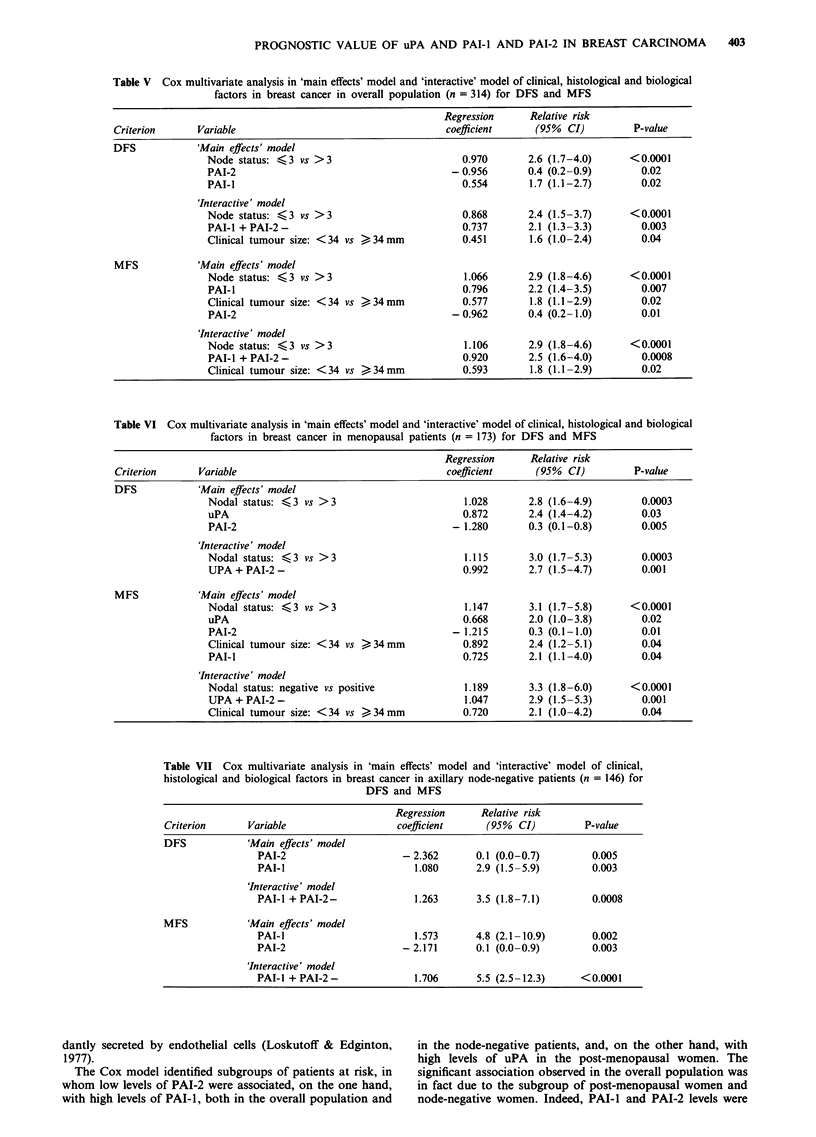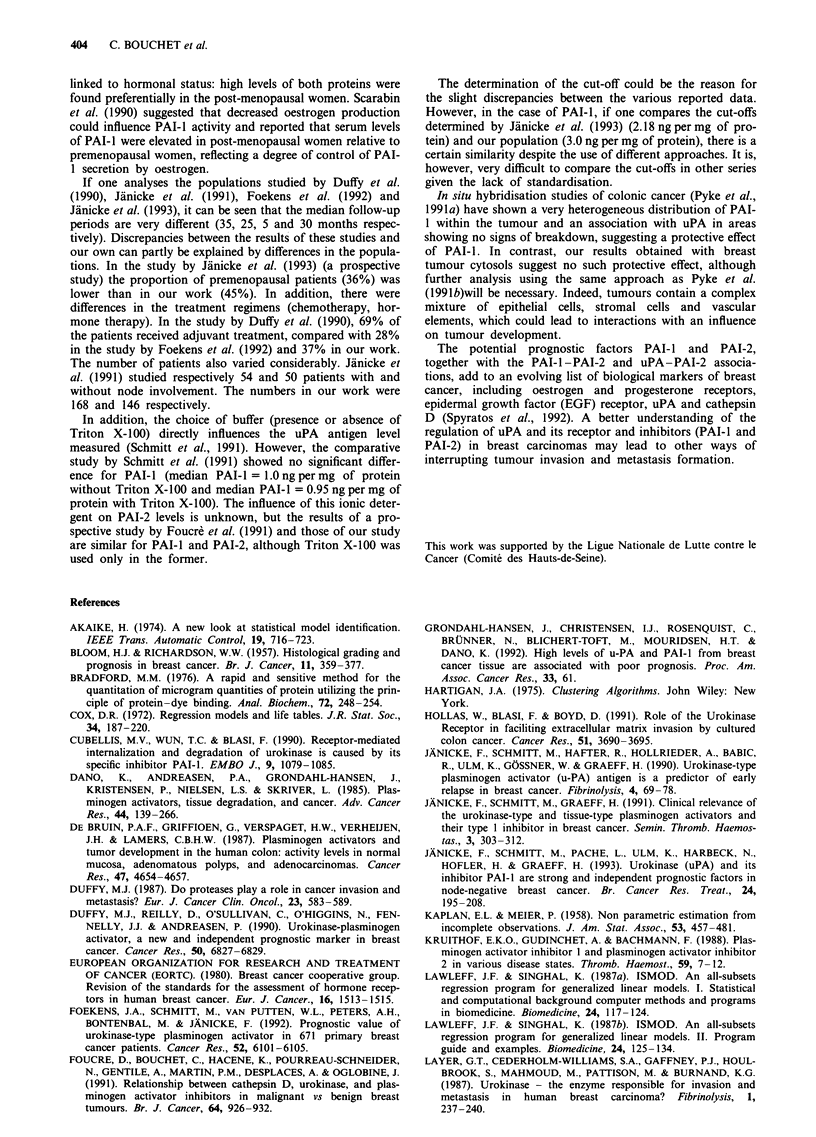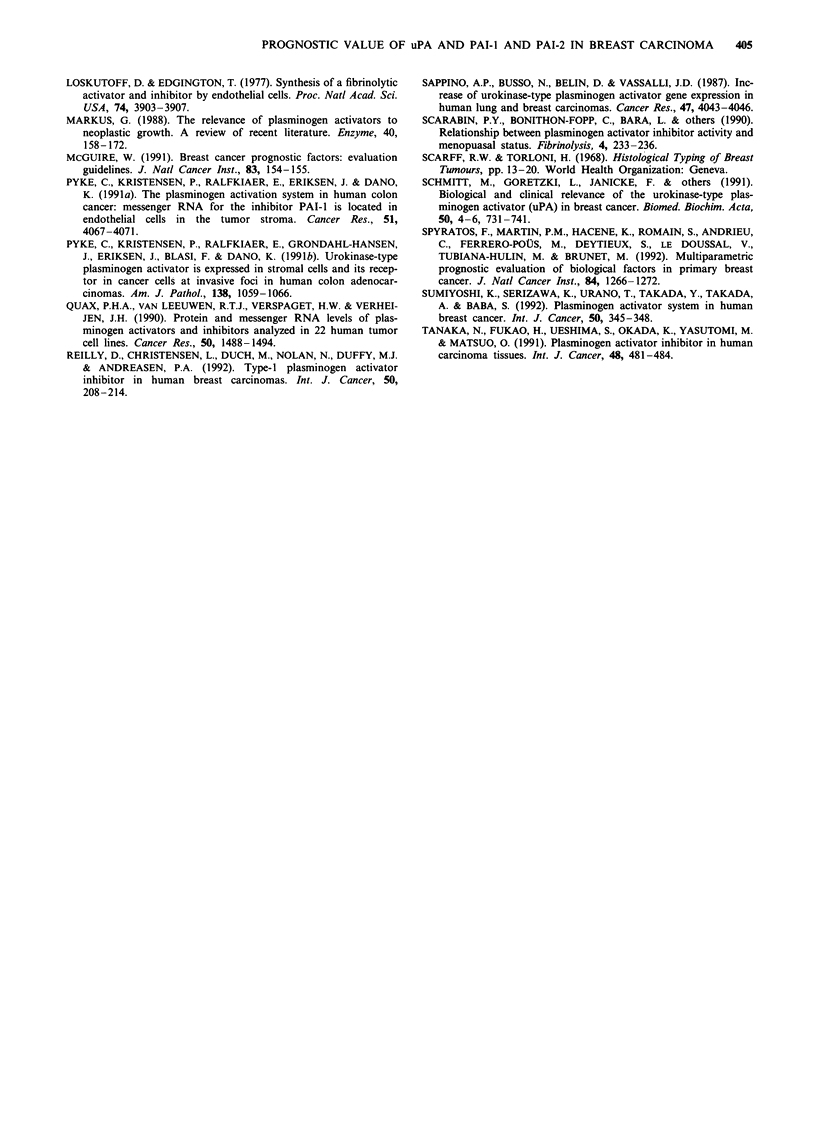# Prognostic value of urokinase-type plasminogen activator (uPA) and plasminogen activator inhibitors PAI-1 and PAI-2 in breast carcinomas.

**DOI:** 10.1038/bjc.1994.74

**Published:** 1994-02

**Authors:** C. Bouchet, F. Spyratos, P. M. Martin, K. Hacène, A. Gentile, J. Oglobine

**Affiliations:** Laboratoire d'immunochimie, Centre René Huguenin, Saint-Cloud, France.

## Abstract

It is now clearly established that proteolytic enzymes, including plasminogen activator (uPA), play an important role in breaking down the extracellular matrix, which is considered to be a step in metastasis formation. Plasminogen activators are controlled at various levels. Two inhibitors, PAI-1 and PAI-2, have been identified, the latter being more specific for uPA. In attempts to determine their prognostic value, it is essential to investigate the relative importance of these parameters and their interactions. We used an immunoenzymatic method to assay uPA, PAI-1 and PAI-2 antigens in cytosols prepared from 314 primary breast tumours. The patients were followed up for a minimum of 6 years and all relevant clinical and laboratory findings were recorded. Univariate analysis confirmed the poor outcome of patients whose tumours contained large amounts of uPA and PAI-1. In addition, low levels of PAI-2 correlated with shorter disease-free survival in the overall population (P = 0.02), post-menopausal women (P = 0.02) and women without lymph node involvement (P = 0.02). Multivariate analysis in the 'main effects' Cox model identified node involvement, macroscopic tumour size and PAI-2 as significant variables. The 'interactive' model, taking into account interactions between uPA and its two inhibitors, identified a first subgroup with a very poor prognosis associating either high levels of PAI-1 with low levels of PAI-2 in the overall population and the women with no node involvement or high levels of uPA with low levels of PAI-2 in the group of menopausal women. We conclude that PAI-1 provides the same prognostic information as uPA, and does not appear to play a role as an inhibitor. In contrast, PAI-2 increases the prognostic value of uPA, particularly in post-menopausal women, and PAI-1 in patients with no node involvement.


Br. J. Cancer (1994), 69, 398–405 © Macmillan Press Ltd., 1994

### Prognostic value of urokinase-type plasminogen activator (uPA) and plasminogen activator inhibitors PAI-1 and PAI-2 in breast carcinomas

C. Bouchet[1], F. Spyratos[2], P.M. Martin[3], K. Hacène[4], A. Gentile[5] & J. Oglobine[1]

[1]*Laboratoire d'immunochimie and* [2]*Laboratoire de biologie tissulaire, Centre René Huguenin, 35 rue Dailly, 92211 Saint-Cloud, France;* [3]*Laboratoire de cancérologie expérimentale, Faculté de Médecine Nord, Boulevard Pierre Dramard, 13326 Marseille, France;* [4]*Département de statistiques médicales and* [5]*Laboratoire d'anatomie-pathologique Centre René Huguenin, 35 rue Dailly, 92211 Saint-Cloud, France.*

**Summary** It is now clearly established that proteolytic enzymes, including plasminogen activator (uPA), play an important role in breaking down the extracellular matrix, which is considered to be a step in metastasis formation. Plasminogen activators are controlled at various levels. Two inhibitors, PAI-1 and PAI-2, have been identified, the latter being more specific for uPA. In attempts to determine their prognostic value, it is essential to investigate the relative importance of these parameters and their interactions. We used an immunoenzymatic method to assay uPA, PAI-1 and PAI-2 antigens in cytosols prepared from 314 primary breast tumours. The patients were followed up for a minimum of 6 years and all relevant clinical and laboratory findings were recorded. Univariate analysis confirmed the poor outcome of patients whose tumours contained large amounts of uPA and PAI-1. In addition, low levels of PAI-2 correlated with shorter disease-free survival in the overall population ($P = 0.02$), post-menopausal women ($P = 0.02$) and women without lymph node involvement ($P = 0.02$). Multivariate analysis in the 'main effects' Cox model identified node involvement, macroscopic tumour size and PAI-2 as significant variables. The 'interactive' model, taking into account interactions between uPA and its two inhibitors, identified a first subgroup with a very poor prognosis associating either high levels of PAI-1 with low levels of PAI-2 in the overall population and the women with no node involvement or high levels of uPA with low levels of PAI-2 in the group of menopausal women. We conclude that PAI-1 provides the same prognostic information as uPA, and does not appear to play a role as an inhibitor. In contrast, PAI-2 increases the prognostic value of uPA, particularly in post-menopausal women, and PAI-1 in patients with no node involvement.

Tumour cell invasion and metastasis formation is a multifactorial process. The remodelling it involves requires the coordinated action of cell-secreted proteolytic enzymes and their inhibitors. Elevated levels of urokinase-type plasminogen activator (uPA) have been implicated in these invasive processes (Dano *et al.*, 1985; Duffy, 1987; Layer *et al.*, 1987; Markus *et al.*, 1988; Pyke *et al.*, 1991b), and plasminogen activator inhibitor type 1 (PAI-1) has been found in many types of malignant tissue (Kruithof *et al.*, 1988; Cubellis *et al.*, 1990; Foucré *et al.*, 1991; Tanaka *et al.*, 1991; Reilly *et al.*, 1992). Plasminogen activator inhibitor type 2 (PAI-2) is one of the primary physiological inhibitors of uPA. Individual prognostic values have been reported in breast cancer (Duffy *et al.*, 1990; Jänicke *et al.*, 1990, 1993; Grondahl-Hansen *et al.*, 1992; Spyratos *et al.*, 1992; Sumiyoshi *et al.*, 1992). The purpose of this study was to analyse the antigen levels of uPA, PAI-1 and PAI-2 by means of an enzyme-linked immunosorbent assay (ELISA) technique and to evaluate their relative value in predicting disease-free and metastasis-free survival rates of primary breast cancer patients.

### Materials and methods

### *Patients*

The 314 patients in this study (mean age 55 years; range 30–86 years) were selected according to the following criteria: primary, unilateral breast carcinoma and treatment at the Centre René Huguenin between 1980 and 1985. The median follow-up was 7 years (maximum 11 years) at the time of the study. Fifty-nine per cent of patients had undergone a total mastectomy and 41% of patients a partial mastectomy; both groups had undergone axillary lymph node clearance. Two hundred and three patients had received post-operative irradiation. Patients ($n = 115$) regarded as high risk [i.e. those with more than three involved axillary lymph nodes or those with at least one involved axillary lymph node and Scarff, Bloom and Richardson (SBR) (Bloom & Richardson, 1957; Scarff & Torloni, 1968) histological grade III tumours and those aged below 35 years] had received post-operative chemotherapy or hormone therapy. All patients received peroperative thiotepa. Two hundred and ninety-two patients underwent clinical, radiological and laboratory tests every 3 months for the first 2 years and yearly thereafter. At the time of analysis, 91 patients had relapsed (37 had recurrences, 78 had distant metastases); of these, 54 died of breast cancer.

### *Tissue extract samples*

Tumour specimens were obtained at surgery, selected by the pathologist and stored in liquid nitrogen until extraction. Age, SBR histological grade, number of involved lymph nodes (mean number of lymph nodes examined = 14; range 7–40), macroscopic tumour size, menopausal status and oestrogen and progesterone receptor status (EORTC, 1980) were known in every case (Table I). For extraction, tissues pieces of 250–300 mg wet weight were pulverised at 4°C in 10 mM Tris–HCl buffer pH 7.4 containing 1.5 mM EDTA, 0.5 mM dithiothreitol and 10% glycerol. The suspension was centrifuged (100,000 *g* at 4°C for 60 min). The cytosols were collected and stored in liquid nitrogen until use.

### *Assay of uPA, PAI-1 and PAI-2*

Levels of uPA, PAI-1 and PAI-2 antigen were measured in cytosols by an immunoenzymatic method (Biopool Tint Elize, Umea, Sweden). Monoclonal anti-uPA antibody was raised against pro-uPA, two forms of uPA (low and high molecular weight) and uPA bound to plasminogen activator inhibitor type 1 or 2 (PAI-1 or PAI-2). Monoclonal anti-PAI-1 antibody recognises active and inactive forms of PAI-1 and PAI-1 bound to uPA or tPA. Monoclonal anti-PAI-2 antibody recognises low molecular weight PAI-2 (44.6 kDa) and glycosylated high molecular weight PAI-2 (60 kDa). After incubation of cytosols for 2 h at 25°C with agitation,

Correspondence: C. Bouchet.
Received 26 March 1993; and in revised form 5 August 1993.

the second polyclonal antibody labelled with peroxidase was added. Absorbance was measured at 405 nm in a microtitre plate reader (Milenia Kinetic Analyser). Antigen levels (ng $ml^{-1}$) were obtained from standard curves. Protein levels (mean = 1.8 ng $ml^{-1}$) were assayed using the Bradford method (Bradford, 1976; Bio Rad, CA, USA). Results were expressed in ng per mg of protein and assays were performed in triplicate. The Biopool Tint Elize Kit commercial standards for sera were optimised by diluting cytosols (1:6 for the assay of PAI-1 and 1:2 for PAI-2) in phosphate-buffered saline (PBS) solution pH 7.4, EDTA/Tween 20, containing bovine serum albumin (BSA) 1:1,000. The detection limit is about 0.1 ng $ml^{-1}$ for uPA, 2.5 ng $ml^{-1}$ for PAI-1 and 4.0 ng $ml^{-1}$ for PAI-2.

### *Statistical methods*

Differences in the distribution of characteristics among patient subgroups were analysed using the $\chi^2$ test. Spearman correlation coefficients ($\rho$) were computed for pairwise combinations of uPA, PAI-1, and PAI-2. The cut-off points for uPA, PAI-1 and PAI-2 were determined independently of prognosis by the *K*-means clustering method (Hartigan, 1975), which separates patients into clusters so that the within-cluster sum of squares is minimised. For each parameter, actuarial disease-free survival (DFS) and metastasis-free survival (MFS) rates were calculated according to the method of Kaplan and Meier (1958) and compared by the log-rank test. DFS was defined as the time from diagnosis to the detection of the first local relapse or distant metastasis, and MFS was defined as the time from diagnosis to the occurrence of the first distant metastasis (locoregional relapse was excluded) or to the end of the study. A forward stepwise multivariate Cox 'main effects' was used to determine the importance of uPA, PAI-1 and PAI-2 relative to known classical prognostic factors (Cox, 1972). Table I presents the patient characteristics candidates for inclusion in the Cox model. It should be noted that variables with more than two outcome states were recoded using dummy variables. In addition to these variables, the potential for interaction between uPA, PAI-1 and PAI-2 was evaluated in a second Cox model, the 'interactive' model. The two models were then compared using Akaike's criterion (Akaike, 1974; Lawleff & Singhal, 1987*a*,*b*) which gives the best-fit model as the one with minimum AIC (Akaike information criterion), defined as: AIC = −2 log (likelihood) + 2 (number of parameters).

### Results

The distribution of clinical, histological and biological factors in the overall study population is shown in Table I. Addi-

**Table I** Description of the population and results of the univariate prognostic analysis

| *Factor* | *Number of patients* | *Number of events (%)* | P-*values* | *Number of metastases (%)* | P-*values* |
|---|---|---|---|---|---|
| Age | | | | | |
| <50 years | 118 | 34 (29) | 0.8 | 30 (25) | 0.9 |
| 50–64 years | 119 | 37 (31) | | 30 (25) | |
| >64 years | 77 | 20 (26) | | 18 (23) | |
| Menopausal status | | | | | |
| Premenopausal | 141 | 38 (27) | 0.2 | 34 (24) | 0.4 |
| Post-menopausal | 173 | 53 (31) | | 44 (26) | |
| Stage | | | | | |
| I | 55 | 13 (24) | 0.04 | 10 (18) | 0.02 |
| II | 219 | 61 (28) | | 52 (24) | |
| II | 40 | 17 (42) | | 16 (40) | |
| Clinical tumour size (mm) | | | | | |
| <34 | 157 | 35 (22) | 0.005 | 27 (17) | 0.001 |
| ⩾34 | 157 | 56 (35) | | 51 (32) | |
| Macroscopic tumour size (mm) | | | | | |
| <29 | 221 | 56 (25) | 0.01 | 46 (21) | 0.01 |
| ⩾29 | 93 | 35 (38) | | 32 (34) | |
| Path. axillary lymph nodes | | | | | |
| 0 | 146 | 32 (22) | <0.0005 | 24 (16) | <0.0005 |
| 1–3 | 98 | 25 (25) | | 21 (21) | |
| >3 | 70 | 34 (48) | | 33 (47) | |
| Scarff, Bloom and Richardson grade | | | | | |
| I | 30 | 6 (20) | 0.5 | 4 (13) | 0.3 |
| II | 199 | 61 (31) | | 51 (26) | |
| III | 85 | 24 (28) | | 23 (27) | |
| Oestrogen receptor level | | | | | |
| <10 fmol $mg^{-1}$ protein | 95 | 29 (30) | 0.6 | 28 (29) | 0.2 |
| ⩾10 fmol $mg^{-1}$ protein | 219 | 62 (28) | | 50 (23) | |
| Progesterone receptor level | | | | | |
| <10 fmol $mg^{-1}$ protein | 137 | 45 (33) | 0.2 | 40 (29) | 0.1 |
| ⩾10 fmol $mg^{-1}$ protein | 177 | 46 (26) | | 38 (21) | |
| uPA status | | | | | |
| uPA − ⩽0.52 ng $mg^{-1}$ protein | 214 | 55 (25) | 0.06 | 46 (21) | 0.03 |
| uPA + >0.52 ng $mg^{-1}$ protein | 100 | 36 (35) | | 32 (32) | |
| PAI-1 status | | | | | |
| PAI-I − ⩽3 ng $mg^{-1}$ protein | 231 | 60 (26) | 0.04 | 49 (21) | 0.007 |
| PAI-1 + >3 ng $mg^{-1}$ protein | 83 | 31 (37) | | 29 (35) | |
| PAI-2 status | | | | | |
| PAI-2 − ⩽14.5 ng $mg^{-1}$ protein | 271 | 85 (31) | 0.02 | 73 (27) | 0.04 |
| PAI-1 + >14.5 ng $mg^{-1}$ protein | 43 | 6 (14) | | 5 (12) | |
| Combination | | | | | |
| PAI-1 − PAI-2 − | 205 | 56 (27) | 0.004 | 46 (22) | 0.001 |
| PAI-1 − PAI-2 + | 26 | 4 (15) | | 3 (11) | |
| PAI-1 + PAI-2 − | 66 | 29 (44) | | 27 (41) | |
| PAI-1 + PAI-2 + | 17 | 2 (12) | | 2 (12) | |

**Table II** Distribution of uPA, PAI-1 and PAI-2 in the overall population of breast cancer patients

| *Variable* | *Median* | *Mean* | *Standard deviation* | *Range* | *Cut-off* |
|---|---|---|---|---|---|
| uPA (ng mg$^{-1}$ protein) | 0.32 | 0.48 | 0.53 | 0–4.4 | 0.52 |
| PAI-1 (ng mg$^{-1}$ protein) | 1.86 | 2.60 | 2.66 | 0–28.9 | 3 |
| PAI-2 (ng mg$^{-1}$ protein) | 2.72 | 8.70 | 19.18 | 0–171 | 14.5 |

tional details on the biological factors are presented in Table II. The cut-off points were 0.52 ng per mg of protein for uPA, 3 ng per mg of protein for PAI-1 and 14.5 ng per mg of protein for PAI-2.

*Relation between uPA, PAI-1, PAI-2 and clinical and histological factors*

uPA was not related to any clinical or histological variable. High levels of uPA were related to high levels of PAI-1 and PAI-2 ($P<0.0001$, $\rho=0.47$, and $P<0.001$, $\rho=0.35$, respectively). High levels of PAI-1 and PAI-2 were related to post-menopausal status ($P=0.008$ and $P<0.01$ respectively). Higher levels of PAI-1 were related to SBR histological grade II and III ($P<0.01$) and showed borderline significance with high values of PAI-2 ($P=0.06$) (not shown).

*Univariate prognostic analysis in the overall population (Table I)*

High levels of uPA showed borderline significance with DFS ($P=0.06$) and were associated with MFS ($P=0.03$). High levels of PAI-1 were associated with significantly shorter DFS ($P=0.04$) and MFS ($P=0.007$) (Figure 1a). In contrast, high values of PAI-2 were associated with longer DFS ($P=0.02$) (Figure 1b) and MFS ($P=0.04$). When PAI-1 and PAI-2 were combined, high levels of PAI-1 and low levels of PAI-2 correlated with poor DFS ($P=0.004$) and MFS ($P=0.001$) (Figure 1c).

*Univariate prognostic analysis according to menopausal status (Table III)*

In premenopausal patients, uPA, PAI-1 and PAI-2 levels were not related to DFS or MFS. In post-menopausal patients, high levels of uPA showed borderline significance with DFS ($P=0.06$) and were associated with shorter MFS ($P=0.04$). High levels of PAI-1 were only associated with shorter MFS ($P=0.02$) (Figure 2a). In contrast, patients with low levels of PAI-2 had shorter DFS ($P=0.02$) and MFS ($P=0.03$) (Figure 2b). The combination of high levels of PAI-1 and low levels of PAI-2 correlated with shorter DFS ($P=0.002$) and MFS ($P=0.001$) (Figure 2c).

*Univariate prognostic analysis according to axillary node status (Table IV)*

In node-postive patients, uPA, PAI-1 and PAI-2 were not related to DFS or MFS. In node-negative patients, uPA was not correlated with DFS or MFS. However, high levels of PAI-1 were associated with shorter DFS ($P=0.02$) and MFS ($P=0.0008$). High levels of PAI-2 were only associated with longer DFS. The combination of high levels of PAI-1 and low levels of PAI-2 was related to shorter DFS ($P=0.0008$) (Figure 3a) and MFS ($P=0.00005$) (Figure 3b).

*Cox multivariate analysis of clinical and histological factors and uPA, PAI-1 and PAI-2 in the overall population (Table V)*

The two models took into account the main effects and interactions respectively. With regard to DFS, the 'main effects' model identified, in the following order, node involvement, PAI-2 and PAI-1 as independent variables. The 'interactive' model increased the individual prognostic value

**Figure 1** Metastasis-free survival as a function of **a**, PAI-1; **b**, PAI-2; and **c**, their association in the overall population. **a**, Patients with high PAI-1 levels had a significantly lower rate of MFS than those with low levels. **b**, Patients with high PAI-2 levels had a significantly higher rate of MFS than those with low levels. **c**, A particularly favourable prognostic group was identified by an association between low PAI-1 and high PAI-2 levels.

**Table III** Univariate prognostic analysis according to DFS and MFS in post-menopausal patients ($n = 173$)

| | | DFS | | | MFS | | |
|---|---|---|---|---|---|---|---|
| *Variable* | *Number of patients* | *Number of events (%)* | *Relative risk* | P-*value* | *Number of metastases (%)* | *Relative risk* | P-*value* |
| UPA | | | | | | | |
| $\leqslant 0.52$ ng mg$^{-1}$ protein | 119 | 31 (26) | 1.0 | 0.06 | 25 (21) | 1.00 | 0.04 |
| $>0.52$ ng mg$^{-1}$ protein | 54 | 22 (41) | 1.70 | | 19 (35) | 1.86 | |
| PAI-1 | | | | | | | |
| $\leqslant 3$ ng mg$^{-1}$ protein | 117 | 31 (26) | 1.00 | 0.1 | 24 (20) | 1.00 | 0.02 |
| $>3$ ng mg$^{-1}$ protein | 56 | 22 (39) | 1.56 | | 20 (36) | 1.91 | |
| PAI-2 | | | | | | | |
| $\leqslant 14.5$ ng mg$^{-1}$ protein | 142 | 49 (35) | 1.00 | 0.02 | 41 (29) | 1.00 | 0.03 |
| $>14.5$ ng mg$^{-1}$ protein | 31 | 4 (13) | 0.32 | | 3 (9) | 0.30 | |
| Combination | | | | | | | |
| PAI-1 − PAI-2 − | 100 | 28 (28) | 1.00 | 0.002 | 22 (22) | 1.00 | 0.001 |
| PAI-1 − PAI-2 + | 17 | 3 (18) | 0.62 | | 2 (12) | 0.56 | |
| PAI-1 + PAI-2 − | 42 | 21 (50) | 2.12 | | 19 (45) | 2.54 | |
| PAI-1 + PAI-2 + | 14 | 1 (7) | 0.20 | | 1 (7) | 0.27 | |

**Table IV** Univariate prognostic analysis according to DFS and MFS in axillary node-negative patients ($n = 146$)

| | | DFS | | | MFS | | |
|---|---|---|---|---|---|---|---|
| *Variable* | *Number of patients* | *Number of events (%)* | *Relative risk* | P-*value* | *Number of metastases (%)* | *Relative risk* | P-*value* |
| UPA | | | | | | | |
| $\leqslant 0.52$ ng mg$^{-1}$ protein | 97 | 18 (18) | 1.00 | NS | 12 (12) | 1.00 | NS |
| $>0.52$ ng mg$^{-1}$ protein | 49 | 14 (28) | 1.59 | | 12 (24) | 2.12 | |
| PAI-1 | | | | | | | |
| $\leqslant 3$ ng mg$^{-1}$ protein | 102 | 17 (16) | 1.00 | 0.02 | 10 (10) | 1.00 | 0.0008 |
| $>3$ ng mg$^{-1}$ protein | 44 | 15 (34) | 2.22 | | 14 (32) | 3.64 | |
| PAI-2 | | | | | | | |
| $\leqslant 14.5$ ng mg$^{-1}$ protein | 121 | 31 (26) | 1.00 | 0.02 | 23 (19) | 1.00 | NS |
| $>14.5$ ng mg$^{-1}$ protein | 25 | 1 (4) | 0.13 | | 1 (4) | 0.19 | |
| Combination | | | | | | | |
| PAI-1 − PAI-2 − | 90 | 17 (19) | 1.00 | 0.0008 | 10 (11) | 1.00 | 0.00005 |
| PAI-1 − PAI-2 + | 12 | 0 (0) | 0 | | 0 (0) | 0 | |
| PAI-1 + PAI-2 − | 31 | 14 (45) | 2.85 | | 13 (42) | 4.66 | |
| PAI-1 + PAI-2 + | 13 | 1 (8) | 0.36 | | 1 (8) | 0.64 | |

of PAI-1 and PAI-2 by identifying a subpopulation with a particularly poor prognosis, in which elevated PAI-1 levels were associated with low PAI-2 levels. With regard to MFS, the main effects model identified, in the following order, node involvement, PAI-1, clinical tumour size and PAI-2 as independent variables. The 'interactive' model identified a subpopulation with a particularly poor prognosis, in which elevated PAI-1 levels were associated with low PAI-2 levels, as for DFS. According to Akaike's criterion, the interactive model was equivalent to the 'main effects' model.

### *Cox multivariate analysis of clinical and histological factors and uPA, PAI-1 and PAI-2 according to menopausal status (Table VI)*

In the premenopausal patients, macroscopic tumour size was the only variable associated with DFS ($P = 0.005$). For MFS, the only independent variable was node involvement ($P = 0.004$) (not shown).

In the post-menopausal patients, the 'main effects' model identified, in the following order, node involvement, uPA and PAI-2 as independent variables for DFS. When interactions were taken into account, the prognostic value of uPA and PAI-2 was increased, identifying a subpopulation with a particularly poor prognosis, in which high levels of uPA were associated with low levels of PAI-2. With regard to MFS, the 'main effects' model identified, in the following order, uPA, PAI-2, clinical tumour size and PAI-1 as independent variables. The 'interactive' model increased the individual prognostic value of uPA and PAI-2, identifying a subpopulation with a particularly poor prognosis, in which high levels of uPA were associated with low levels of PAI-2.

### *Cox multivariate analysis of clinical and histological factors and uPA, PAI-1 and PAI-2 according to axillary node status (Table VII)*

In the patients with node involvement, only the number of affected nodes was selected by the Cox model, both for DFS ($P = 0.0008$) and for MFS ($P = 0.0002$). This also held true for DFS ($P = 0.0008$) when the interactions between the relevant variables were taken into account. The 'interactive' model identified an MFS population in which low levels of PAI-1 were associated with low levels of uPA ($P = 0.05$); this minimised the pejorative effect of node involvement, which remained the most important factor ($P = 0.0002$) (not shown).

In the patients free of lymph node involvement, the 'main effects' model identified PAI-2 and PAI-1 as independent variables for MFS and DFS. Similarly, when interactions were taken into account in the DFS and MFS models, the prognostic value of PAI-2 and PAI-1 was increased; a subpopulation was identified in which the prognosis was particularly poor and in which high levels of PAI-1 were associated with low levels of PAI-2.

**Figure 2** Metastasis-free survival as a function of **a**, PAI-1; **b**, PAI-2; and **c**, their association in post-menopausal women. **a**, Patients with high PAI-1 levels had a significantly lower rate of MFS than those with low levels. **b**, Patients with high PAI-2 levels had a significantly higher rate of MFS than those with low levels. **c**, A particularly favourable prognostic group was identified by an association between high PAI-1 and high PAI-2 levels.

**Figure 3** **a**, Disease-free survival; and **b**, Metastasis-free survival as a function of the association of PAI-1 and PAI-2 in axillary node-negative women. DFS and MFS were particularly good in women with low PAI-1 and high PAI-2 levels.

### Discussion

The involvement of uPA in the invasion and metastatic mechanisms is well documented (De Bruin *et al.*, 1987; Sappino *et al.*, 1987; Jänicke *et al.*, 1990; Quax *et al.*, 1990; Hollas *et al.*, 1991), but the exact role of the two inhibitors PAI-1 and PAI-2 is less well known.

To better analyse their prognostic value, it is important to evaluate the relative importance of these three parameters and their interactions. To this end, we measured uPA, PAI-1 and PAI-2 antigen levels in a series of 314 breast cancer specimens and studied their relationships with classic parameters, as well as their prognostic value.

We confirmed that uPA and PAI-1 were independent of classical prognostic factors (Duffy *et al.*, 1990; Jänicke *et al.*, 1990; Grondhal-Hansen *et al.*, 1992; Reilly *et al.*, 1992), as well as the strong relationship between uPA and PAI-1 (Reilly *et al.*, 1992). We also confirmed the poor prognosis of patients with tumours containing high levels of uPA and PAI-1 (Duffy *et al.*, 1990; Jänicke *et al.*, 1990; Grondhal-Hansen *et al.*, 1992; Spyratos *et al.*, 1992; Sumiyoshi *et al.*, 1992). In contrast, high levels of PAI-2 were associated with a favourable prognosis not only in the overall population, but also in the subgroup of post-menopausal women and those with no node involvement. PAI-2, which has highest affinity constant for uPA, appears to be a true inhibitor, contrary to PAI-1. Sumiyoshi *et al.* (1992) reported that PAI-2 levels were higher in node-negative patients, but did not provide a prognostic analysis.

The correlation between uPA and PAI-1 antigen levels suggests one of two possibilities (Reilly *et al.*, 1992): either PAI-1 is a defence mechanism against tumoral invasion that has been inactivated at the time of analysis, or it plays a role in plasminogen activation. Our results, showing a link between high levels of PAI-1 and a poor outcome, tend to support the second hypothesis and to confirm the redundancy of the information provided by PAI-1 and uPA. PAI-1 could also be a marker of neovascularisation, as it is abun-

**Table V** Cox multivariate analysis in 'main effects' model and 'interactive' model of clinical, histological and biological factors in breast cancer in overall population ($n = 314$) for DFS and MFS

| *Criterion* | *Variable* | *Regression coefficient* | *Relative risk (95% CI)* | P-*value* |
|---|---|---|---|---|
| DFS | *'Main effects' model* | | | |
| | Node status: ⩽3 *vs* >3 | 0.970 | 2.6 (1.7–4.0) | <0.0001 |
| | PAI-2 | – 0.956 | 0.4 (0.2–0.9) | 0.02 |
| | PAI-1 | 0.554 | 1.7 (1.1–2.7) | 0.02 |
| | *'Interactive' model* | | | |
| | Node status: ⩽3 *vs* >3 | 0.868 | 2.4 (1.5–3.7) | <0.0001 |
| | PAI-1 + PAI-2 – | 0.737 | 2.1 (1.3–3.3) | 0.003 |
| | Clinical tumour size: <34 *vs* ⩾34 mm | 0.451 | 1.6 (1.0–2.4) | 0.04 |
| MFS | *'Main effects' model* | | | |
| | Node status: ⩽3 *vs* >3 | 1.066 | 2.9 (1.8–4.6) | <0.0001 |
| | PAI-1 | 0.796 | 2.2 (1.4–3.5) | 0.007 |
| | Clinical tumour size: <34 *vs* ⩾34 mm | 0.577 | 1.8 (1.1–2.9) | 0.02 |
| | PAI-2 | – 0.962 | 0.4 (0.2–1.0) | 0.01 |
| | *'Interactive' model* | | | |
| | Node status: ⩽3 *vs* >3 | 1.106 | 2.9 (1.8–4.6) | <0.0001 |
| | PAI-1 + PAI-2 – | 0.920 | 2.5 (1.6–4.0) | 0.0008 |
| | Clinical tumour size: <34 *vs* ⩾34 mm | 0.593 | 1.8 (1.1–2.9) | 0.02 |

**Table VI** Cox multivariate analysis in 'main effects' model and 'interactive' model of clinical, histological and biological factors in breast cancer in menopausal patients ($n = 173$) for DFS and MFS

| *Criterion* | *Variable* | *Regression coefficient* | *Relative risk (95% CI)* | P-*value* |
|---|---|---|---|---|
| DFS | *'Main effects' model* | | | |
| | Nodal status: ⩽3 *vs* >3 | 1.028 | 2.8 (1.6–4.9) | 0.0003 |
| | uPA | 0.872 | 2.4 (1.4–4.2) | 0.03 |
| | PAI-2 | – 1.280 | 0.3 (0.1–0.8) | 0.005 |
| | *'Interactive' model* | | | |
| | Nodal status: ⩽3 *vs* >3 | 1.115 | 3.0 (1.7–5.3) | 0.0003 |
| | UPA + PAI-2 – | 0.992 | 2.7 (1.5–4.7) | 0.001 |
| MFS | *'Main effects' model* | | | |
| | Nodal status: ⩽3 *vs* >3 | 1.147 | 3.1 (1.7–5.8) | <0.0001 |
| | uPA | 0.668 | 2.0 (1.0–3.8) | 0.02 |
| | PAI-2 | – 1.215 | 0.3 (0.1–1.0) | 0.01 |
| | Clinical tumour size: <34 *vs* ⩾34 mm | 0.892 | 2.4 (1.2–5.1) | 0.04 |
| | PAI-1 | 0.725 | 2.1 (1.1–4.0) | 0.04 |
| | *'Interactive' model* | | | |
| | Nodal status: negative *vs* positive | 1.189 | 3.3 (1.8–6.0) | <0.0001 |
| | UPA + PAI-2 – | 1.047 | 2.9 (1.5–5.3) | 0.001 |
| | Clinical tumour size: <34 *vs* ⩾34 mm | 0.720 | 2.1 (1.0–4.2) | 0.04 |

**Table VII** Cox multivariate analysis in 'main effects' model and 'interactive' model of clinical, histological and biological factors in breast cancer in axillary node-negative patients ($n = 146$) for DFS and MFS

| *Criterion* | *Variable* | *Regression coefficient* | *Relative risk (95% CI)* | P-*value* |
|---|---|---|---|---|
| DFS | *'Main effects' model* | | | |
| | PAI-2 | – 2.362 | 0.1 (0.0–0.7) | 0.005 |
| | PAI-1 | 1.080 | 2.9 (1.5–5.9) | 0.003 |
| | *'Interactive' model* | | | |
| | PAI-1 + PAI-2 – | 1.263 | 3.5 (1.8–7.1) | 0.0008 |
| MFS | *'Main effects' model* | | | |
| | PAI-1 | 1.573 | 4.8 (2.1–10.9) | 0.002 |
| | PAI-2 | – 2.171 | 0.1 (0.0–0.9) | 0.003 |
| | *'Interactive' model* | | | |
| | PAI-1 + PAI-2 – | 1.706 | 5.5 (2.5–12.3) | <0.0001 |

dantly secreted by endothelial cells (Loskutoff & Edginton, 1977).

The Cox model identified subgroups of patients at risk, in whom low levels of PAI-2 were associated, on the one hand, with high levels of PAI-1, both in the overall population and in the node-negative patients, and, on the other hand, with high levels of uPA in the post-menopausal women. The significant association observed in the overall population was in fact due to the subgroup of post-menopausal women and node-negative women. Indeed, PAI-1 and PAI-2 levels were linked to hormonal status: high levels of both proteins were found preferentially in the post-menopausal women. Scarabin *et al.* (1990) suggested that decreased oestrogen production could influence PAI-1 activity and reported that serum levels of PAI-1 were elevated in post-menopausal women relative to premenopausal women, reflecting a degree of control of PAI-1 secretion by oestrogen.

If one analyses the populations studied by Duffy *et al.* (1990), Jänicke *et al.* (1991), Foekens *et al.* (1992) and Jänicke *et al.* (1993), it can be seen that the median follow-up periods are very different (35, 25, 5 and 30 months respectively). Discrepancies between the results of these studies and our own can partly be explained by differences in the populations. In the study by Jänicke *et al.* (1993) (a prospective study) the proportion of premenopausal patients (36%) was lower than in our work (45%). In addition, there were differences in the treatment regimens (chemotherapy, hormone therapy). In the study by Duffy *et al.* (1990), 69% of the patients received adjuvant treatment, compared with 28% in the study by Foekens *et al.* (1992) and 37% in our work. The number of patients also varied considerably. Jänicke *et al.* (1991) studied respectively 54 and 50 patients with and without node involvement. The numbers in our work were 168 and 146 respectively.

In addition, the choice of buffer (presence or absence of Triton X-100) directly influences the uPA antigen level measured (Schmitt *et al.*, 1991). However, the comparative study by Schmitt *et al.* (1991) showed no significant difference for PAI-1 (median PAI-1 = 1.0 ng per mg of protein without Triton X-100 and median PAI-1 = 0.95 ng per mg of protein with Triton X-100). The influence of this ionic detergent on PAI-2 levels is unknown, but the results of a prospective study by Foucrè *et al.* (1991) and those of our study are similar for PAI-1 and PAI-2, although Triton X-100 was used only in the former.

The determination of the cut-off could be the reason for the slight discrepancies between the various reported data. However, in the case of PAI-1, if one compares the cut-offs determined by Jänicke *et al.* (1993) (2.18 ng per mg of protein) and our population (3.0 ng per mg of protein), there is a certain similarity despite the use of different approaches. It is, however, very difficult to compare the cut-offs in other series given the lack of standardisation.

*In situ* hybridisation studies of colonic cancer (Pyke *et al.*, 1991*a*) have shown a very heterogeneous distribution of PAI-1 within the tumour and an association with uPA in areas showing no signs of breakdown, suggesting a protective effect of PAI-1. In contrast, our results obtained with breast tumour cytosols suggest no such protective effect, although further analysis using the same approach as Pyke *et al.* (1991*b*)will be necessary. Indeed, tumours contain a complex mixture of epithelial cells, stromal cells and vascular elements, which could lead to interactions with an influence on tumour development.

The potential prognostic factors PAI-1 and PAI-2, together with the PAI-1–PAI-2 and uPA–PAI-2 associations, add to an evolving list of biological markers of breast cancer, including oestrogen and progesterone receptors, epidermal growth factor (EGF) receptor, uPA and cathepsin D (Spyratos *et al.*, 1992). A better understanding of the regulation of uPA and its receptor and inhibitors (PAI-1 and PAI-2) in breast carcinomas may lead to other ways of interrupting tumour invasion and metastasis formation.

This work was supported by the Ligue Nationale de Lutte contre le Cancer (Comité des Hauts-de-Seine).

### References

AKAIKE, H. (1974). A new look at statistical model identification. *IEEE Trans. Automatic Control*, **19**, 716–723.

BLOOM, H.J. & RICHARDSON, W.W. (1957). Histological grading and prognosis in breast cancer. *Br. J. Cancer*, **11**, 359–377.

BRADFORD, M.M. (1976). A rapid and sensitive method for the quantitation of microgram quantities of protein utilizing the principle of protein–dye binding. *Anal. Biochem.*, **72**, 248–254.

COX, D.R. (1972). Regression models and life tables. *J.R. Stat. Soc.*, **34**, 187–220.

CUBELLIS, M.V., WUN, T.C. & BLASI, F. (1990). Receptor-mediated internalization and degradation of urokinase is caused by its specific inhibitor PAI-1. *EMBO J.*, **9**, 1079–1085.

DANO, K., ANDREASEN, P.A., GRONDAHL-HANSEN, J., KRISTENSEN, P., NIELSEN, L.S. & SKRIVER, L. (1985). Plasminogen activators, tissue degradation, and cancer. *Adv. Cancer Res.*, **44**, 139–266.

DE BRUIN, P.A.F., GRIFFIOEN, G., VERSPAGET, H.W., VERHEIJEN, J.H. & LAMERS, C.B.H.W. (1987). Plasminogen activators and tumor development in the human colon: activity levels in normal mucosa, adenomatous polyps, and adenocarcinomas. *Cancer Res.*, **47**, 4654–4657.

DUFFY, M.J. (1987). Do proteases play a role in cancer invasion and metastasis? *Eur. J. Cancer Clin. Oncol.*, **23**, 583–589.

DUFFY, M.J., REILLY, D., O'SULLIVAN, C., O'HIGGINS, N., FENNELLY, J.J. & ANDREASEN, P. (1990). Urokinase-plasminogen activator, a new and independent prognostic marker in breast cancer. *Cancer Res.*, **50**, 6827–6829.

EUROPEAN ORGANIZATION FOR RESEARCH AND TREATMENT OF CANCER (EORTC). (1980). Breast cancer cooperative group. Revision of the standards for the assessment of hormone receptors in human breast cancer. *Eur. J. Cancer.*, **16**, 1513–1515.

FOEKENS, J.A., SCHMITT, M., VAN PUTTEN, W.L., PETERS, A.H., BONTENBAL, M. & JÄNICKE, F. (1992). Prognostic value of urokinase-type plasminogen activator in 671 primary breast cancer patients. *Cancer Res.*, **52**, 6101–6105.

FOUCRE, D., BOUCHET, C., HACENE, K., POURREAU-SCHNEIDER, N., GENTILE, A., MARTIN, P.M., DESPLACES, A. & OGLOBINE, J. (1991). Relationship between cathepsin D, urokinase, and plasminogen activator inhibitors in malignant *vs* benign breast tumours. *Br. J. Cancer*, **64**, 926–932.

GRONDAHL-HANSEN, J., CHRISTENSEN, I.J., ROSENQUIST, C., BRÜNNER, N., BLICHERT-TOFT, M., MOURIDSEN, H.T. & DANO, K. (1992). High levels of u-PA and PAI-1 from breast cancer tissue are associated with poor prognosis. *Proc. Am. Assoc. Cancer Res.*, **33**, 61.

HARTIGAN, J.A. (1975). *Clustering Algorithms*. John Wiley: New York.

HOLLAS, W., BLASI, F. & BOYD, D. (1991). Role of the Urokinase Receptor in faciliting extracellular matrix invasion by cultured colon cancer. *Cancer Res.*, **51**, 3690–3695.

JÄNICKE, F., SCHMITT, M., HAFTER, R., HOLLRIEDER, A., BABIC, R., ULM, K., GÖSSNER, W. & GRAEFF, H. (1990). Urokinase-type plasminogen activator (u-PA) antigen is a predictor of early relapse in breast cancer. *Fibrinolysis*, **4**, 69–78.

JÄNICKE, F., SCHMITT, M., GRAEFF, H. (1991). Clinical relevance of the urokinase-type and tissue-type plasminogen activators and their type 1 inhibitor in breast cancer. *Semin. Thromb. Haemostas.*, **3**, 303–312.

JÄNICKE, F., SCHMITT, M., PACHE, L., ULM, K., HARBECK, N., HOFLER, H. & GRAEFF, H. (1993). Urokinase (uPA) and its inhibitor PAI-1 are strong and independent prognostic factors in node-negative breast cancer. *Br. Cancer Res. Treat.*, **24**, 195–208.

KAPLAN, E.L. & MEIER, P. (1958). Non parametric estimation from incomplete observations. *J. Am. Stat. Assoc.*, **53**, 457–481.

KRUITHOF, E.K.O., GUDINCHET, A. & BACHMANN, F. (1988). Plasminogen activator inhibitor 1 and plasminogen activator inhibitor 2 in various disease states. *Thromb. Haemost.*, **59**, 7–12.

LAWLEFF, J.F. & SINGHAL, K. (1987*a*). ISMOD. An all-subsets regression program for generalized linear models. I. Statistical and computational background computer methods and programs in biomedicine. *Biomedicine*, **24**, 117–124.

LAWLEFF, J.F. & SINGHAL, K. (1987*b*). ISMOD. An all-subsets regression program for generalized linear models. II. Program guide and examples. *Biomedicine*, **24**, 125–134.

LAYER, G.T., CEDERHOLM-WILLIAMS, S.A., GAFFNEY, P.J., HOULBROOK, S., MAHMOUD, M., PATTISON, M. & BURNAND, K.G. (1987). Urokinase – the enzyme responsible for invasion and metastasis in human breast carcinoma? *Fibrinolysis*, **1**, 237–240.

LOSKUTOFF, D. & EDGINGTON, T. (1977). Synthesis of a fibrinolytic activator and inhibitor by endothelial cells. *Proc. Natl Acad. Sci. USA*, **74**, 3903–3907.

MARKUS, G. (1988). The relevance of plasminogen activators to neoplastic growth. A review of recent literature. *Enzyme*, 40, 158–172.

McGUIRE, W. (1991). Breast cancer prognostic factors: evaluation guidelines. *J. Natl Cancer Inst.*, **83**, 154–155.

PYKE, C., KRISTENSEN, P., RALFKIAER, E., ERIKSEN, J. & DANO, K. (1991*a*). The plasminogen activation system in human colon cancer: messenger RNA for the inhibitor PAI-1 is located in endothelial cells in the tumor stroma. *Cancer Res.*, **51**, 4067–4071.

PYKE, C., KRISTENSEN, P., RALFKIAER, E., GRONDAHL-HANSEN, J., ERIKSEN, J., BLASI, F. & DANO, K. (1991*b*). Urokinase-type plasminogen activator is expressed in stromal cells and its receptor in cancer cells at invasive foci in human colon adenocarcinomas. *Am. J. Pathol.*, **138**, 1059–1066.

QUAX, P.H.A., VAN LEEUWEN, R.T.J., VERSPAGET, H.W. & VERHEIJEN, J.H. (1990). Protein and messenger RNA levels of plasminogen activators and inhibitors analyzed in 22 human tumor cell lines. *Cancer Res.*, **50**, 1488–1494.

REILLY, D., CHRISTENSEN, L., DUCH, M., NOLAN, N., DUFFY, M.J. & ANDREASEN, P.A. (1992). Type-1 plasminogen activator inhibitor in human breast carcinomas. *Int. J. Cancer*, **50**, 208–214.

SAPPINO, A.P., BUSSO, N., BELIN, D. & VASSALLI, J.D. (1987). Increase of urokinase-type plasminogen activator gene expression in human lung and breast carcinomas. *Cancer Res.*, **47**, 4043–4046.

SCARABIN, P.Y., BONITHON-FOPP, C., BARA, L. & others (1990). Relationship between plasminogen activator inhibitor activity and menopuasal status. *Fibrinolysis*, **4**, 233–236.

SCARFF, R.W. & TORLONI, H. (1968). *Histological Typing of Breast Tumours*, pp. 13–20. World Health Organization: Geneva.

SCHMITT, M., GORETZKI, L., JANICKE, F. & others (1991). Biological and clinical relevance of the urokinase-type plasminogen activator (uPA) in breast cancer. *Biomed. Biochim. Acta*, **50**, 4–6, 731–741.

SPYRATOS, F., MARTIN, P.M., HACENE, K., ROMAIN, S., ANDRIEU, C., FERRERO-POÜS, M., DEYTIEUX, S., LE DOUSSAL, V., TUBIANA-HULIN, M. & BRUNET, M. (1992). Multiparametric prognostic evaluation of biological factors in primary breast cancer. *J. Natl Cancer Inst.*, **84**, 1266–1272.

SUMIYOSHI, K., SERIZAWA, K., URANO, T., TAKADA, Y., TAKADA, A. & BABA, S. (1992). Plasminogen activator system in human breast cancer. *Int. J. Cancer*, **50**, 345–348.

TANAKA, N., FUKAO, H., UESHIMA, S., OKADA, K., YASUTOMI, M. & MATSUO, O. (1991). Plasminogen activator inhibitor in human carcinoma tissues. *Int. J. Cancer*, **48**, 481–484.